# Inlays—Points for the Beginner

**Published:** 1906-03

**Authors:** E. P. Beadles

**Affiliations:** Danville, Va.


### INLAYS—POINTS FOR THE BEGINNER.

By E. P. Beadles, D. D. S., Danville, Va.

In this work, as in all other, good judgment must be exercised. For the beginner only cavities in the anterior upper teeth should be filled with porcelain. I shall speak of proximal cavities only. The first thing to be considered should be

SPACE.

There are several ways by which to get room enough to work. In porcelain inlay work possibly a little more space is needed than for the gold filling; but very little more. The cavity should be cut out slightly from the lingual surface and a piece of dry cotton tightly wedged. This should be left for twenty-four hours. If the teeth are not wide enough apart by this time the process should be repeated. This second wedging may make them a little sore, hence it might be well to leave the second piece in for forty eight hours, if there is no hurry, when the soreness will have passed away. If perfectly convenient to see the patient, the cotton may be removed when space enough has been gotten and a little gutta percha put in until the soreness passes away. My own experience is that this process of wedging seldom makes the teeth tender enough to delay the operation.

PREPARATION OF CAVITY.

Much has been written upon this subject, but the beginner need not be afraid, only a little common sense and ordinary skill are necessary. Cut away freely the lingual wall, exposing the entire cavity to view in the mirror (all of this work of the anterior upper teeth should be done by reflected light). Of course there should be no undercuts, the edges should be smoothly polished, and shaped at right angles.

Take a piece of platimum foil one thousandth of an inch in thickness and slide it between the teeth, one edge reaching up beyond the free margin of the gum. The size of the platinum to be in accordance with size of the cavity (i. e., large enough to leave an extended edge after filling the cavity), with the plyers carry a pledget of wet cotton to the foil and gently press to place. Then use ball-pointed burnisher for thoroughly adapting the cotton. Remove the pledget and use only the ball burnisher until the foil is perfectly adapted to the walls, all creases smoothed out, then with a flat burnisher press the foil to the edges. As a final adaptation the wet cotton may again be used.

No attention need be paid to a tear in the bottom of the metal matrix, but if the edge should be torn where it laps over and beyond the cavity, a new trial must be made. If not, the porcelain will flow into the space and prevent a fit, or the edge of the torn place will be caught in the fusing and refuse to come out thus preventing a perfect fitting of the inlay, or causing a dark spot.

### FUSING.

I generally use the high-fusing material, but the low-fusing out-fit should be in place, as there are times when it will be needed. Grasp the platinum impression (no investment is necessary) with a pair of wire spring plyers and put in the mixed powder, filtered river water or distilled water will answer, filling the cavity in the platinum about full, dry out on the apron of furnace. This should be thorough or the steam will blow out the powder. A half minute or more will fuse this sufficiently if the temperature is right. Remove, core off and add more mix until the proper contour is reached. Two or three fusings may be necessary. With a little practice the eye will tell you the temperature of the furnace and this must be the guide as to the length of time the bake should be left in. Be careful not to over-fuse, as the edges of the inlay will be rough and a bad fit will result. The inlay is then dipped in water and the foil stripped off.

### UNDERCUTS.

A small wheel bur may be used to make sufficient undercuts, and to roughen the cavity. A diamond disk, not too thin, should be used to cut proper grooves in the inlay, when it may be cemented to place.

### COLOR.

For the beginner, making inlays such as above described, the shade guides may be depended upon for colors, always remembering, however, that the coloring may be burned out. For these proximal fillings the shade should be somewhat lighter than the tooth appears.

### CEMENT.

This, as well as the color, is too great a subject to be treated in an article like this. After the beginner has gotten as far as the above indicates, he will find plently of literature for further guidance. I have here set forth the work in its simplest form, and in all things the simpler the better. No one need be afraid to make the attempt. It saves much wear upon both operator and patient, to say nothing of other advantages.

